# Implications of DNA Methylation in Parkinson’s Disease

**DOI:** 10.3389/fnmol.2017.00225

**Published:** 2017-07-18

**Authors:** Ernesto Miranda-Morales, Karin Meier, Ada Sandoval-Carrillo, José Salas-Pacheco, Paola Vázquez-Cárdenas, Oscar Arias-Carrión

**Affiliations:** ^1^Unidad de Trastornos del Movimiento y Sueño, Hospital General Dr. Manuel Gea González Mexico City, Mexico; ^2^Instituto de Investigación Científica, Universidad Juárez del Estado de Durango Durango, Mexico; ^3^Departamento de Genética Molecular, Instituto de Fisiología Celular, Universidad Nacional Autónoma de México Mexico City, Mexico; ^4^Clínica de Obesidad, Hospital General Dr. Manuel Gea González Mexico City, Mexico

**Keywords:** 5-methylcytosine, DNA methylation, epigenetics, folate, alpha-synuclein, neurodegeneration, Parkinson disease

## Abstract

It has been 200 years since Parkinson’s disease (PD) was first described, yet many aspects of its etiopathogenesis remain unclear. PD is a progressive and complex neurodegenerative disorder caused by genetic and environmental factors including aging, nutrition, pesticides and exposure to heavy metals. DNA methylation may be altered in response to some of these factors; therefore, it is proposed that epigenetic mechanisms, particularly DNA methylation, can have a fundamental role in gene–environment interactions that are related with PD. Epigenetic changes in PD-associated genes are now widely studied in different populations, to discover the mechanisms that contribute to disease development and identify novel biomarkers for early diagnosis and future pharmacological treatment. While initial studies sought to find associations between promoter DNA methylation and the regulation of associated genes in PD brain tissue, more recent studies have described concordant DNA methylation patterns between blood and brain tissue DNA. These data justify the use of peripheral blood samples instead of brain tissue for epigenetic studies. Here, we summarize the current data about DNA methylation changes in PD and discuss the potential of DNA methylation as a potential biomarker for PD. Additionally, we discuss environmental and nutritional factors that have been implicated in DNA methylation. Although the search for significant DNA methylation changes and gene expression analyses of PD-associated genes have yielded inconsistent and contradictory results, epigenetic modifications remain under investigation for their potential to reveal the link between environmental risk factors and the development of PD.

## Introduction

Parkinson’s disease (PD) is the second most common chronic neurodegenerative disease in the elderly population. The motor symptoms that characterize PD are bradykinesia, tremor, rigidity, and postural instability, together with non-motor symptoms such as depression, anxiety, sleep disorders and cognitive dysfunction. These symptoms lead to severe impairment of the quality of life for the PD patient (Frucht, 2004). Pathological analyses of *post-mortem* brains have shown Lewy bodies, which are abnormal protein aggregates found within nerve cells, and a progressive loss of *substantia nigra* dopamine neurons (Iacono et al., 2015). Among the molecular mechanisms suggested to cause PD are cellular oxidative stress and autophagy. Pesticide exposure, use of well water, heavy metal exposure, and industrialization are some of the environmental factors that contribute to the development of PD (Willis et al., 2010a,b).

Over recent years, extensive genetic screening of PD families has aimed at identifying mutations associated with the disease that would give a deeper insight into the molecular mechanisms underlying the PD pathology. Genetic studies identified several genomic risk *loci* associated with familial PD, such as *PARK1-15* and other related genes (Masliah et al., 2000; Cheon et al., 2012). Additionally, other genes, including *LRRK2*, *SNCA*, *MAPT*, and *GBA*, have been associated with sporadic PD (Coppede, 2012). Even though there is evidence that PD can be caused sporadically by familial genetic mutations (causal mutations), such as in *alpha-synuclein* (*SNCA*) or *Parkin*, it is more likely that in most patients the disease develops as a consequence of the combination of mutations in multiple PD-associated genes and environmental risk factors.

In addition to the genetic component involved in the development of many disorders (acquired mutations in one gene or a group of genes), epigenetic mechanisms have been found to contribute significantly to their development. Epigenetic factors are chemical modifications of chromatin or its regulatory proteins that do not change the underlying genomic sequence. These modifications can modulate gene expression, allowing differentiation into different cellular phenotypes by driving tissue-specific expression patterns. These changes include DNA methylation, post-translational modifications of histones, chromatin remodeling, as well as small and long non-coding RNAs (Turner, 2007).

Epigenetic regulation of biological processes is known to be essential during embryonic development, early brain programming, neurogenesis and brain plasticity (Yao et al., 2016). Therefore, it is not surprising that epigenetic deregulation can be critical for the onset of various neurodegenerative diseases, such as PD (Ammal Kaidery et al., 2013). Previously, a comprehensive genomic study identified several PD risk *loci* in cerebellum and frontal cortex of PD brains, including *PARK16*, *GPNMB*, and *STX1B* genes, that were associated with differential DNA methylation at proximal CpG sites (International Parkinson’s Disease Genomics Consortium (IPDGC) and Wellcome Trust Case Control Consortium 2 (WTCCC2), 2011).

Importantly, as there is currently no animal model that mimics PD; human brain is still the model used to study epigenetic changes. However, as PD brain tissue is predominantly analyzed *post-mortem*, this data cannot provide information about disease-progressive alterations, the extent of variations induced by previous therapeutic treatments and the occurrence of potential PD biomarkers. Access to brain samples for research is limited, so the focus has been on finding a more easily accessible tissue, such as peripheral blood, as a surrogate for brain tissue. For this purpose, a genome-wide study examined DNA methylation changes in PD patients by collecting fresh *post-mortem* brain and blood samples from PD patients and age-matched healthy subjects (Masliah et al., 2013). This comparison revealed that both tissues exhibited highly similar global DNA methylation patterns. Accordingly, Masliah et al. (2013) identified groups of genes with either increased or decreased DNA methylation in both PD brain and blood samples. Importantly, analysis of DNA methylation profiles of blood clearly distinguishes PD patients from healthy subjects or subjects with other disorders. These results suggest that, firstly, peripheral blood may be a valid surrogate for brain tissue samples, and secondly, epigenetic changes could potentially serve as biomarkers for the diagnosis of PD. Early biomarkers could improve the prognosis of PD by facilitating the initiation of rational treatment before significant neurological damage takes place. Here, we discuss the current evidence for DNA methylation changes in PD, including the involvement of nutrition and environmental factors.

## The Role of DNA Methylation in Disease and Aging

DNA methylation is the most studied epigenetic modification, one that has been investigated in almost all pathologies. In mammals, DNA methylation takes place predominantly in the context of CpG dinucleotides (Ehrlich and Wang, 1981). While overall the genome is widely depleted of CpGs, CpG islands are regions of high CpG content (Takai and Jones, 2002; Wu et al., 2010). CpG islands are characteristic for more than 60% of all promoters of protein-coding genes. Whereas on a genome-wide level up to 70–80% of all CpG sites are methylated, CpG islands are mostly devoid of DNA methylation (Bird, 2002; Edwards et al., 2010). Adjacent to a CpG island, 2 kilo base pairs (kb) up- and down-stream, are its so-called CpG shores (Irizarry et al., 2009). The presence of DNA methylation, at least at gene promoters and regions of repetitive sequences, is linked to chromatin silencing (Hsieh, 1994; Siegfried et al., 1999). Two principal mechanisms, which are not mutually exclusive, are thought to explain the repressive effect of DNA methylation on gene repression (Bird and Wolffe, 1999; Klose and Bird, 2006). First: DNA methylation interferes with the recognition of transcription factor (TF) binding sites and thereby impairs gene activation (Domcke et al., 2015). Second: DNA methylation is recognized by specific Methyl-CpG binding proteins, such as MeCP2, that recruit co-repressor protein complexes and thereby mediate silencing (Nan et al., 1998). Aberrant methylation patterns at CpG islands and shores have been linked to human disease, including multiple cancers (Ohm et al., 2007; Irizarry et al., 2009; Berman et al., 2011; Manjegowda et al., 2017).

In developing embryos and germ cells, DNA methylation patterns are first established by *de novo* DNA methyltransferases (DNMTs), DNMT3A and DNMT3B. After this, DNA methylation is maintained during DNA replication by DNMT1, which localizes to the replication fork during S-phase where it binds to hemimethylated CpGs (Jones and Liang, 2009; Jung et al., 2017). In 2009, ten-eleven translocation (TET) enzymes were identified that can reverse DNA methylation, by oxidization of 5-methyl cytosine (5mC) to 5-hydroxymethyl cytosine (5hmC) (Tahiliani et al., 2009; Pastor et al., 2013). 5hmC can be lost passively by dilution during replication or be actively removed by subsequent oxidative reactions catalyzed by TET proteins that result in the formation of 5-formylcytosine (5fC) and 5-carboxylcytosine (5caC) as intermediates (Ito et al., 2011). Finally, the thymine DNA glycosylase (TDG)-mediated base excision repair (BER) replaces the methylated site by an unmodified cytosine (He et al., 2011; Kohli and Zhang, 2013).

Importantly, it appears that the 5hmC modification is not only an intermediate of 5mC demethylation, but has been considered to be an epigenetic mark in itself. Although the exact role of 5hmC is still being studied intensively, it seems to have a distinct function from that of 5mC. Particularly in neuronal cells, DNA hydroxymethylation was found to be enriched in gene bodies of actively transcribed genes (Mellen et al., 2012; Hahn et al., 2013). Given the relative abundance of DNA hydroxymethylation in the brain and its apparent role in normal brain maturation and memory formation (Szulwach et al., 2011; Kaas et al., 2013; Lister et al., 2013; Kinde et al., 2015), it has been implicated in the onset and progression of several neurodegenerative disorders (Villar-Menendez et al., 2013; Wang et al., 2013; Condliffe et al., 2014; Coppieters et al., 2014). Although not as widespread as 5mC and 5hmC, there is emerging evidence that cytosine methylation also exists outside of the sequence context of CpG sites (non-CpG methylation: CpA, CpT, and CpC) and appears to be most common in embryonic stem cells (Lister et al., 2009) and adult brain tissue (Varley et al., 2013; Guo et al., 2014). Non-CpG methylation occurs postnatally during the primary phase of neuronal maturation and may play a role in transcriptional repression (Lister et al., 2013; Guo et al., 2014). However, as it is technically challenging to target this modification *in vivo* without altering CpG methylation in the process, extensive research is still required to elucidate the distinct biological function of non-CpG methylation.

Genetically, aging is characterized by distinct alterations that take place at the chromatin level. These include telomere shortening, increased genome instability and changes of epigenetic signatures, such as DNA methylation patterns (Lopez-Otin et al., 2013). Age-related remodeling of DNA methylation comprises events of both hypo- and hypermethylation (Maegawa et al., 2010; Jung and Pfeifer, 2015). DNA hypomethylation happens globally at CpG sites outside of CpG islands (Christensen et al., 2009; Heyn et al., 2012; Day et al., 2013), while DNA hypermethylation affects mostly CpG islands in promoters of genes, which are frequently involved in development and differentiation (Christensen et al., 2009; Rakyan et al., 2010). Therefore, over time the accumulation of epimutations, which are heritable changes of gene activity mediated by epigenetic alterations, are believed to contribute to genomic instability just as genetic mutations do.

Interestingly, the methylation states at specific CpG *loci* can be consulted as epigenetic biomarkers to reliably predict the human chronological age (Horvath, 2013; Weidner et al., 2014). Moreover, twin studies determined that in each human, the age-dependent aggregation of distinct epigenetic changes, termed ‘epigenetic drift,’ is thought to be influenced predominantly by environmental factors (Fraga et al., 2005; Tan Q. et al., 2016). The individual differences in exposure to these factors are suspected to contribute to variation in disease susceptibility, onset, progression, etiopathology, treatment response and disease outcome. Taken together, changes in DNA methylation patterns and their effects on chromatin and gene expression appear to add increasingly to our understanding of age-related diseases, including PD. In the following, we will summarize and discuss the current evidence of DNA methylation changes at candidate genes that could be related to the development of PD.

## DNA Methylation and PD: Analysis of the *SNCA* Gene

The *SNCA*p.Ala53Thr mutation, described in 1997, was the first genetic cause of PD identified (Polymeropoulos et al., 1997). This missense mutation provided the first link between *SNCA* and familial PD after its identification in a family from Southern Italy. The respective gene product, the SNCA protein, was discovered almost simultaneously (Spillantini et al., 1997). At the molecular level, SNCA aggregation contributes majorly to the formation of Lewy bodies, a hallmark of PD pathology. In addition to genetic mutations, also *SNCA locus* amplifications (duplications, triplications) have been found as a cause of familial PD (Singleton et al., 2003; Chartier-Harlin et al., 2004). *SNC*A point mutations, as well as gene multiplications and overexpression, are all thought to play a causal role in the formation of Lewy bodies (Narhi et al., 1999; Masliah et al., 2000). That *SNCA* gene dosage is critical for the development of PD, was further supported by mouse models with neuronal expression of wild-type *SNCA* (Masliah et al., 2000; Janezic et al., 2013). These transgenic mice revealed PD-like loss of dopaminergic neurons, protein aggregate formation, and motor impairments.

As gene dosage can be changed not only by gene amplification, but also by gene regulation, DNA methylation was considered as a potential mechanism that could be involved in the deregulation of *SNCA* in the case of PD. Accordingly, sequence analysis of the promoter region of the *SNCA* gene led to the identification of two CpG islands (Matsumoto et al., 2010). The first, CpG-1 is located in the first exon but does not overlap with the coding region of *SNCA*, and the second, CpG-2 is located in the first intron. In luciferase reporter assays the promoter activity of sequences containing CpG-2 was indeed strongly reduced by *in vitro* DNA methylation prior to cell transfection (Jowaed et al., 2010; Matsumoto et al., 2010). Furthermore, treatment of SK-N-SH cells with a DNA methylation inhibitor resulted in a reduction of CpG-2 methylation and a significant increase of *SNCA* mRNA and protein levels (Jowaed et al., 2010). These data supported the idea that DNA methylation at least at the intronic CpG-2 island could control *SNCA* gene activity. In fact, several studies analyzing the DNA methylation levels in samples of PD patients compared to controls confirmed a hypomethylation of intron 1 that coincides with the second *SNCA* CpG island. Using brain samples a significant demethylation of intron 1 was reported in the *substantia nigra pars compacta* (SnPC) of PD patients which could explain increased *SNCA* expression (Jowaed et al., 2010; Matsumoto et al., 2010). Taking into account the observation that DNA methylation patterns between blood and brain tissue show a strong correlation (Masliah et al., 2013), more recent studies analyzed peripheral blood samples of PD subjects instead of or in addition to *post-mortem* brain tissue. In agreement with the assumption that DNA methylation profiles in the brain could be potentially mirrored in blood cells, a recent study found *SNCA* promoter hypomethylation in both *post-mortem* cortex and peripheral blood samples (Pihlstrom et al., 2015). Another study reported hypomethylation of *SNCA* intron 1 in peripheral blood mononuclear cells of 100 sporadic PD subjects (Ai et al., 2014). In 2015, the largest study carried out to date analyzing 490 peripheral blood samples of patients with sporadic PD, also revealed hypomethylation of *SNCA* intron 1. In contrast, *SNCA* methylation was found to be increased in PD patients who received higher L-dopa dosage. Accordingly, L-dopa led to a specific increment of DNA methylation of *SNCA* intron 1 in mononuclear cell cultures. Interestingly, the detection of DNMT1 in *post-mortem* brain tissue of PD patients and in *SNCA* transgenic mice uncovered that the amount of enzyme was strongly reduced in the nuclear fraction of neuronal cells (Desplats et al., 2011). Thus, sequestration of DNMT1 in the cytosol could explain the global, as well as the *SNCA* gene-specific, PD-dependent DNA hypomethylation, mechanistically.

**FIGURE 1:**
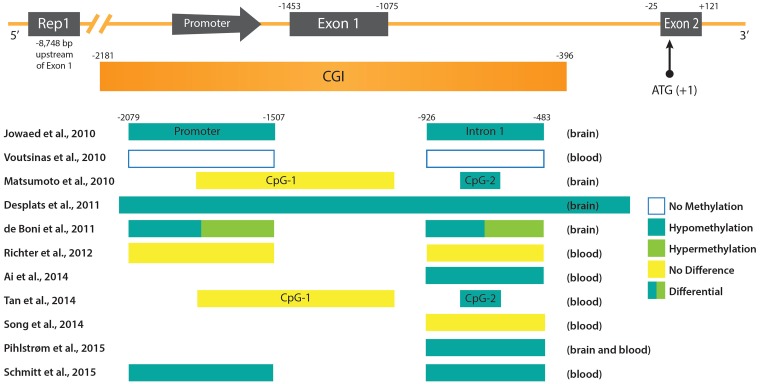
DNA methylation patterns in PD at the promoter region of the *SNCA* gene. **(Upper)** The promoter region and 5′ end of the *SNCA* gene containing the core promoter and the two first exons of *SNCA* (Exon 1 and Exon 2) are depicted. Exon 2 contains the ATG start codon of the open reading frame (ORF). Upstream of the promoter the location of the polymorphic microsatellite sequence Rep1 is shown. Below the scheme of the *SNCA* gene the CpG-rich region is depicted as an CpG island (CGI). Positions of regulatory sequences (promoter and CGIs), as well as exons and introns are given at the top in reference to the ATG start codon. **(Lower)** Regulatory sequences and intronic regions analyzed in various studies (see text for more detail) are depicted as boxes. The references are given on the left, and the DNA methylation status and source of PD samples on the right.

In recent years, meta-analyses of genome-wide association studies (GWAS) on single nucleotide polymorphism (SNP) data of large PD case-control cohorts were conducted (Nalls et al., 2011, 2014; Sharma et al., 2012). These studies identified risk *loci* in both genes, previously not linked to PD pathology, and known key players, such as *SNCA*. Thereby, obtained results substantiated that there is a major genetic component contributing to the susceptibility to PD. But additionally, inter-individual genetic variants can frequently be associated with DNA methylation differences at distinct CpG sites and are defined in statistical analyses as methylation quantitative trait *loci* (mQTLs). Two recent studies investigated the relationship between genetic variation and CpG methylation in the human brain (Gibbs et al., 2010; Zhang et al., 2010). In case of the *SNCA* gene, three independent studies noted that the genotype SNP rs3756063 showed a significant correlation with the DNA methylation state of *SNCA* intron 1 both in brain and blood PD samples (Pihlstrom et al., 2015; Schmitt et al., 2015; Wei et al., 2016). However, it should be noted that an association between rs3756063 and the *SNCA* mRNA expression could not be found (Pihlstrom et al., 2015; Wei et al., 2016). Furthermore, another association could be established between the *SNCA* DNA methylation levels and the Rep1 polymorphism (Ai et al., 2014). In contrast to rs3756063, Rep1 is a complex microsatellite repeat polymorphism located approximately 10 kb upstream of the *SNCA* transcription start site (**Figure 1**). Its longest 263 bp allele has previously been associated with sporadic PD (Maraganore et al., 2006). In agreement with an elevated PD risk, genotypes carrying the 263 bp allele showed the strongest *SNCA* intron 1 hypomethylation (Ai et al., 2014). Experiments in transgenic mice suggested a *cis*-regulatory effect of the Rep1-length regulating *SNCA* transcription, whereby homozygosity of the expanded 263 bp allele correlated with the highest gene expression (Cronin et al., 2009). Controversially, recent data obtained by the clustered regularly interspaced short palindromic repeats (CRISPR)/Cas9 technique, to edit the genome Rep1 *locus* in human embryonic stem cell-derived neurons, contradict the enhancer function of the repeat sequence and do not detect a correlation between *SNCA* expression and Rep1-length (Soldner et al., 2016). Likewise, the interdependency between DNA methylation alterations in the *SNCA locus* and genetic variants, is not understood mechanistically, neither in case of Rep1 nor rs3756063. Genetic variation does not only impact DNA methylation, but also dictates differences in binding of TFs in individuals on a genome-wide level (Kasowski et al., 2010). Therefore, it will be a great challenge to unravel the molecular mechanisms behind these associations, as they are expected to be connected with each other in a complicated network.

Although the studies cited above suggest DNA hypomethylation of the *SNCA* promoter region in PD patients, discrepancies with other findings exist. For example, one study revealed variations of DNA methylation levels at the *SNCA* gene in different brain regions. Both hypomethylation, as well as hypermethylation, were detected in various Lewy body disease/PD stages in both the promoter region and intron 1 (de Boni et al., 2011). Another recent study found no intron 1 hypomethylation of *SNCA* in a limited number of PD patients (Guhathakurta et al., 2017). Moreover, the analysis of blood samples from 43 PD subjects provided no evidence for DNA methylation changes within the *SNCA* promoter region (Richter et al., 2012), just as another study comprising blood leukocyte samples of 50 PD patients (Song et al., 2014). However, it should be noted, that in the latter two studies (Richter et al., 2012; Song et al., 2014) 10 times less subjects participated compared to the analysis of Schmitt et al. (2015) that detected differential DNA methylation at the *SNCA* gene.

In light of the inconsistencies concerning the relevance of DNA hypomethylation at the *SNCA* intron 1 in association with PD, additional studies will be needed to resolve these doubts. Also, even if *SNCA* intron 1 hypomethylation can be consistently confirmed in PD, similar DNA patterns were also found in both Dementia with Lewy Bodies (DLB) (Funahashi et al., 2017) and AD (Yoshino et al., 2016). Therefore, it is conceivable that this DNA methylation change would not serve as a specific biomarker for PD, but a more general one for all Lewy body pathologies.

## DNA Methylation Patterns at PD-Associated Genes

In the following section, we will give an overview of what is known so far about the epigenetic signatures of DNA methylation at genes that were found to be associated with the development of PD (compare **Table 1**).

**Table 1:** DNA methylation status of PD associated genes.

Gene	Alias	Location	DNA methylation	Reference
*PAD2*	Peptidyl arginine deiminase 2	1p36.13	No difference	Mastronardi et al., 2007
*ATP13A2*	*PARK9*	1p36.13	No difference	Behrens et al., 2010
*DJ-1*	*PARK7*	1p36.23	No methylation	Tan Y. et al., 2016
*NPAS2*	Neuronal PAS2	2q11.2	Hypomethylation	Lin et al., 2012
*UCHL1*	Ubiquitin C-terminal hydrolase L1	4p13	No difference	Barrachina and Ferrer, 2009
*PGC1-*α	Peroxisome proliferator-activated receptor gamma coactivator 1-alpha	4p15.2	Hypermethylation	Su et al., 2015
*TNF-*α	Tumor necrosis factor-alpha	6p21.33	No difference	Pieper et al., 2008
*PARK2*	Parkinson juvenile disease protein 2, *Parkin*	6q26	No difference	Cai et al., 2011
			No difference	De Mena et al., 2013
*CYP2E1*	Cytochrome P450-J	10q26.3	Hypomethylation	Kaut et al., 2012
*NOS2*	Nitric oxide synthase 2	17q11.2	Hypomethylation	Searles Nielsen et al., 2015
*MAPT*	Microtubule associated protein Tau	17q21.31	Differential methylation	Coupland et al., 2014
*FANCC/TNKS2*	Fanconi anemia group C protein/tankyrase 2	9q22.32/10q23.32	Differential methylation	Moore et al., 2014
*PARK16/GPNMB/STX1B*	*PARK16/*glycoprotein Nmb*/*syntaxin 1B	1q32/7p15.3/16p11.2	Differential methylation	International Parkinson’s Disease Genomics Consortium [IPDGC], and Wellcome Trust Case Control Consortium 2 [WTCCC2]
Genome-wide	Top 30 differentially methylated genes: *KCTD5, VAV2, MOG, TRI M10, HLA-DQA1, ARHGEF10, GFPT 2, HLA-DRB5, TMEM9, MRI 1, MAPT, HLA-DRB6, LASS3, GSTTP 2, GSTTP. DNAJA3, JAKMIP 3, FRK, LRR C27, DMBX1, LGALS7, FOXK1, APBA1, MAGI2, SLC25A24, GSTT 1, MYOM2, MIR886, TUBA3E, TMCO3*		Hypermethylation Hypomethylation	Masliah et al., 2013

*From left to right: Listed are genes, gene aliases, genomic locations (according to the latest GRCh38/hg38 assembly of the human genome available at the UCSC genome browser), DNA methylation status in PD and references*.

Due to its relation to *SNCA* and its abundance in neurofibrillary lesions of patients with AD, the *beta-synuclein* (*SNCB*) gene has also been considered as a possible player in PD. Interestingly, SNCB inhibits the generation of SNCA fibril aggregation *in vitro* (Park et al., 2003) and therefore, may play a neuroprotective role (Vigneswara et al., 2013). Furthermore, *SNCB* and *SNCA* have similar expression levels in nervous system tissue samples (Maroteaux et al., 1988). Moreover, SNCA and SNCB associate *in vitro* whereby SNCB may protect SNCA from aging-related protein damage (Vigneswara et al., 2013). However, to date, the specific role SNCB plays in PD has not been elucidated. A DNA methylation study carried out in PD samples to examine the *SNCB* gene found its promoter to be unmethylated in *post-mortem* brain from PD and PD-Dementia samples. Additionally, bisulfite sequencing of the *SNCB* promoter in four pure diffuse Lewy body pathology cases did not reveal methylated cytosines along the CpG island (Beyer et al., 2010).

Post-translational citrullination (deimination) is mediated by peptidyl arginine aminases (PADs) and has been implicated as an unusual pathological trait in neurodegeneration and inflammatory responses in multiple sclerosis, AD and prion diseases (Jang et al., 2013). Alterations in the expression of these proteins have also been seen in *post-mortem* samples taken from different brain areas of PD subjects (Nicholas, 2011). In thymus samples from multiple sclerosis patients, the promoter of *PAD2* (peptidyl arginine deaminase type II) was reported to be hypomethylated (Sokratous et al., 2016). In contrast, white matter from PD, AD, or Huntington disease patients showed that *PAD2* was not hypomethylated (Mastronardi et al., 2007). DNA methylation analysis of the tumor necrosis factor alpha (TNF-alpha) gene, another PD-associated gene, showed a significantly lower methylation level comparing DNA from the SnPC to DNA from brain cortex. However, this difference could not be linked to PD as it was observed in both PD subjects and controls (Pieper et al., 2008). In another study, the *UCHL1* promoter from *post-mortem* frontal cortex samples was analyzed, and no differences in the percentage of CpG methylation between PD cases and controls were found (Barrachina and Ferrer, 2009).

Behrens et al. analyzed the *ATP13A2* promoter region from four PD subjects with Kufor-Rakeb syndrome, a rare Type 9 juvenile PD that is linked to a mutation in the *ATP13A2* gene (Behrens et al., 2010). No significant correlation between DNA methylation changes of the hypomethylated promoter and Kufor-Rakeb syndrome juvenile PD progression was found. Another study considered the known association of *Parkin* (*PARK2*) gene mutations with autosomal recessive juvenile PD. Samples from 17 PD subjects with heterozygous *Parkin* mutations, as well as 17 PD subjects without *Parkin* mutations, were compared to samples from 10 normal subjects. No significant differences in DNA methylation at CpG sites among these three groups were found, suggesting that a DNA methylation-related mechanism involving the *Parkin* gene was unlikely to play a role in the pathogenesis and development of this type of PD (Cai et al., 2011). A recent study compared the DNA methylation status of the *PARK2* promoter region in 5 *post-mortem* brain samples taken from *substantia nigra*, cerebellum, and occipital cortex (De Mena et al., 2013). In agreement, with previous results (Cai et al., 2011) no differential DNA methylation of *PARK2* was seen (De Mena et al., 2013).

The expression of clock genes, which are components of the circadian clock, is altered in leukocytes from patients with PD (Cai et al., 2010). With this in mind, a study was recently carried out in which DNA methylation status of the clock genes *PER1*, *PER2*, *CRY1*, *CRY2*, *Clock*, *NPAS2*, and *BMAL1* was measured in genomic DNA isolated from blood samples of 206 PD subjects. DNA methylation was detectable in *CRY1* and *NPAS2* promoters whereas the remaining gene promoters analyzed were devoid of DNA methylation. Interestingly, DNA methylation frequency of the *NPAS2* promoter was significantly decreased in PD patients, suggesting that its promoter DNA methylation may contribute to the expression of clock genes in PD (Lin et al., 2012). This finding could be relevant, as sleep disturbance is a commonly reported early symptom of PD (Breen et al., 2014).

Another gene of interest for the analysis of epigenetic changes is the microtubule-associated protein tau (*MAPT*) gene, as a genetic association with PD has been noted in GWAS (Simon-Sanchez et al., 2009). When 28 *post-mortem* brain and 358 blood leukocyte samples were analyzed, higher DNA methylation in MAPT was detected in H1 haplotype versus H2 (Coupland et al., 2014). Notably, in previous studies the presence of the H1 haplotype was associated significantly with PD (Kwok et al., 2004; Zabetian et al., 2007; Refenes et al., 2009). Additionally, DNA hypermethylation of the MAPT gene was observed in the cerebellum, but not in putamen from PD subjects where the MAPT gene was hypomethylated as compared with controls (Coupland et al., 2014).

DNA hypermethylation of the peroxisome proliferator-activated receptor gamma coactivator-1 α (PGC-1α) promoter was reported in a sample of sporadic PD *substantia nigra* samples compared to 10 age-matched controls (Su et al., 2015). Recently, *PARK7* (*DJ-1*) DNA methylation was analyzed in peripheral blood leukocytes in PD subjects and controls. In contrast to the hypermethylated PGC-1α promoter (Su et al., 2015), they found the CpG-1 and CpG-2 islands of *PARK7* to be unmethylated in both PD and the negative control group (Tan Y. et al., 2016).

To detect further PD associated DNA methylation variations, an epigenome-wide association study was done to analyze DNA methylation patterns in putamen samples from *post-mortem* brain tissue of six PD patients. DNA methylation levels were quantitatively determined at 27,500 CpG sites representing 14,495 genes. This analysis revealed decreased DNA methylation at the cytochrome P450 2E1 (*CYP2E1*) gene, together with increased expression of the respective *CYP2E1* messenger RNA, suggesting that this cytochrome gene may contribute to PD susceptibility. In another epigenome-wide association study, conducted to reveal prioritized genes and pathways with statistically significant DNA methylation changes in PD, followed by a subsequent replication analysis of top-ranked CpG sites, single CpG sites of *FANCC* and *TNKS2* showed significant differential methylation between PD cases and controls (Moore et al., 2014). In total, 20 unique genes were identified with a sizable difference in DNA methylation.

Despite the lack of conclusive evidence for the involvement of DNA methylation in the epigenetic regulation of many PD-associated genes, the search for other PD-associated genes and their DNA methylation status is ongoing. Undoubtedly, the significance and consistency of results of genomic DNA methylation in promoter regions of blood samples in comparison with brain samples need to be tested further. Unfortunately, findings from studies searching for PD-specific DNA methylation signatures at the *SNCA* gene and other PD-associated genes are still inconsistent concerning the clinical significance and specificity of DNA methylation changes in PD. Most importantly, experimental evidence that directly links DNA methylation changes in PD to the deregulation of these genes is still missing. Thus, the importance of differential DNA methylation for molecular mechanisms contributing to the development of PD remains to be investigated in future studies.

## Nutritional Factors and their Implications for PD

### DNA Methylation and Folate Deficiency

In recent years, several studies have attempted to pinpoint an interrelation between DNA methylation and folate. However, a significant association between DNA methylation levels and folate status could not always be consistently replicated (Waterland and Jirtle, 2003; Waterland et al., 2006; Steegers-Theunissen et al., 2009; Tobi et al., 2009; Shin et al., 2010). Apart from folate, many other nutrients are known to play key roles in one-carbon metabolism and DNA methylation. More accurate studies that analyze the contribution of other nutrients involved in DNA methylation and gene-diet interactions for PD risk are necessary.

The extent of DNA methylation in the cell is directly associated with the physiological level of SAM, the major methyl donor for DNA methylation, and SAH, the demethylation product of SAM and an inhibitor of DNA methyltransferases. The ratio SAM/SAH is interpreted as the methylation potential and is determined by the homocysteine concentration. The latter is considered a biomarker of folate deficiency, as it is dependent on 5-methyl tetrahydrofolate (THF) availability in the one-carbon metabolism. The physiologic levels of homocysteine and subsequent methylation potential are determined primarily by the dietary intakes of methionine, folate, B12 vitamin and other nutrients.

Importantly, elevated levels of homocysteine may have a toxic effect on dopaminergic neurons (de Lau et al., 2005; Gorgone et al., 2012). Consistently, higher homocysteine concentrations have been reported in PD patients compared to controls. Moreover, serum homocysteine levels predict the SAM/SAH ratio in plasma, and the concentration of SAH shows a significant correlation with markers of neurodegeneration (Amyloid Precursor Protein and SNCA). This evidence supports the use of total homocysteine and SAM/SAH ratio as biomarkers of the DNA methylation potential in patients with PD (Obeid et al., 2009). However, we still lack information about the direct effect of nutrient intake on genomic DNA methylation, especially regarding the combinatorial effects of nutrients with other factors, such as gene polymorphisms and/or therapeutic drugs.

### One-Carbon Metabolism and Polymorphisms

The presence of SNPs in genes encoding enzymes and transporters involved in the folate metabolism, impair methyl group bioavailability and have been associated with altered blood concentrations of biochemical markers, including folate, vitamin B12 and homocysteine (Hazra et al., 2009; Tanaka et al., 2009; Liang et al., 2014). Importantly, some SNPs led to changes of homocysteine levels and were associated with differences in global DNA methylation levels (Wernimont et al., 2011). Other SNPs have also been associated with diseases, such as neural tube defects (Carter et al., 2011; Fisk Green et al., 2013; Ouyang et al., 2013; Liu et al., 2014) and different types of cancers (Curtin et al., 2007; Collin et al., 2009; Gibson et al., 2011; Levine et al., 2011; Metayer et al., 2011; Weiner et al., 2012).

The C677T variant (rs1801133), in the gene encoding the enzyme methylene-tetrahydrofolate reductase (MTHFR), is one of the most-studied SNPs occurring in components of the one-carbon metabolism. The base C677T substitution results in an amino acid change in the catalytic domain of the enzyme. This variation leads to a reduced protein stability and a 30% and 65% reduction of enzymatic activity in heterozygotes (CT) and homozygotes (TT), respectively (Rozen, 1997). Notably, the C677T variant has been previously reported to be associated with PD susceptibility (de Lau et al., 2005; Wu et al., 2013). A recent meta-analysis including data from fifteen studies (comprising 2690 PD cases and 8465 controls) did not find an appreciable difference in the general allelic frequency distribution of C677T between PD cases and controls (Zhu et al., 2015). However, in separate analyses that were stratified for ethnicity, a clear association was detected in Europeans (OR = 1.17), but not in Asians. Interestingly, this appears to be in line with the observation that the allelic frequency of the MTHFR C677T variant differs considerably between ethnic groups (Wilcken et al., 2003; Gueant-Rodriguez et al., 2006). Furthermore, this study confirmed that the T allele is an independent risk factor for increased homocysteine levels in PD patients (Zhu et al., 2015). In contrast, the results of a cohort study analyzing Chinese patients suggested that the A-T haplotype of A1298C, another common MTHFR variant, and C677T decreases the PD susceptibility (Yuan et al., 2016). The inconsistent findings for the association between C677T and PD may be explained by different genetic backgrounds, environmental factors or DNA methylation modulation.

## Other Nutritional/Environmental Factors and DNA Methylation

### Coffee Drinking

The risk for PD is ∼25% lower for coffee drinkers with a linear dose-response effect (Costa et al., 2010; Delamarre and Meissner, 2017). Caffeine is thought to act as an adenosine receptor antagonist, and to reduce inflammation and lipid-mediated oxidative stress (Farooqui and Farooqui, 2011; Kolahdouzan and Hamadeh, 2017).

Little is known whether DNA methylation changes can arise in response to distinct coffee consumption patterns. A recent study using blood tissue data of patients without PD found the methylation status of CpG sites located near genes previously linked to some familial forms of PD (*GBA*, *PARK2/Parkin*, and *PINK1*) associated with coffee consumption (Chuang et al., 2017). However, whether distinct DNA methylation levels at these CpG sites in coffee-drinkers are indeed protective against PD, remains to be further investigated.

### Manganese

Manganese (Mn) is an essential element, but some industrial activities can result in exposure to high occupational and environmental Mn levels (Bowler et al., 2011, 2016). In the environment, Mn in drinking-water and foods may also contribute to toxic effects (ATSDR, 2012).

Exposure to excessive amounts of Mn may lead to adverse health outcomes, and evidence suggests that DNA methylation changes induced by Mn may play a relevant role. Regarding PD risk, gene activity of *PARK2* and *PINK1* was altered via DNA hypermethylation in dopaminergic human neuroblastoma SH-SY5Y cells upon Mn exposure (Tarale et al., 2016). Furthermore, mice exposed to MnCl_2_ showed DNA hypo- and hypermethylation of different *loci* in *substantia nigra* (Yang et al., 2016). In human, the effects of Mn on parkinsonism via DNA methylation changes was assessed in welders’ blood samples. Interestingly, subjects recently exposed to welding fume had lower *NOS2* gene DNA methylation than subjects retired from welding worksites. Also, an inverse association between duration of welding fume exposure and DNA methylation of a *NOS2* CpG site was observed (Searles Nielsen et al., 2015).

### Endocrine Disruptors and Pesticides

It is proposed that other factors, such as endocrine disruptors or pesticide exposure, may play a role in modulating DNA methylation, although the evidence from studies with PD patients or animal models is still limited. Results from experimental, clinical, and epidemiological studies implicate exposure to endocrine disruptors with processes related to neurodegenerative diseases (Kajta and Wojtowicz, 2013; Preciados et al., 2016). Among these compounds, Bisphenol-A has been linked to lower levels of DNA methylation in cerebral cortex and hippocampus in mice (Kumar and Thakur, 2017). Several studies have shown an association with frequent pesticide exposure in men and late-onset PD (Delamarre and Meissner, 2017). Recent findings show that organochlorines exposure of hippocampal-primary cultures causes global hypomethylation of DNA (Wnuk et al., 2016).

Although research suggests that these and other environmental exposures can modify epigenetic signatures; important questions remain open. Therefore, studies in this field will provide new insights into PD pathologic processes, and consequently provide novel preventive and therapeutic intervention strategies.

## Concluding Remarks and Perspectives

Currently, there is a plethora of methods used for measuring DNA methylation (Kurdyukov and Bullock, 2016). To date the “gold standard” for the quantification of DNA methylation is still considered to be bisulfite sequencing. Nowadays this method is often used for genome-wide studies in combination with next-generation sequencing. However, the generation of bisulfite-converted DNA, and its subsequent use has often been described as technically challenging. Additionally, bisulfite conversion can lead to DNA fragmentation and can make amplification of long DNA regions difficult while resulting in chimeric products (Kurdyukov and Bullock, 2016). An easier method is needed that does not require bisulfite conversion, for example, an endonuclease digestion-based assay (historically the first technique utilized for studying DNA methylation), which can be applied at gene-specific *loci*, but is also compatible with whole genome methylation profiling. Determining a standardized method for quantifying DNA methylation at the same genomic regions of reported PD-associated genes would be ideal for clinical research. Another issue is finding a method that can efficiently detect 5hmC and distinguish it from 5mC, not only on a genome-wide level, but also at bp resolution. Techniques applied to analyze DNA methylation changes associated with PD have mostly not discriminated between 5mC and 5hmC. Particularly in the brain, it will be of great interest to unravel whether the latter DNA methylation mark exhibits disease-specific patterns that could serve as biomarkers. A recent study found an approximate two-fold increase of global DNA hydroxymethylation in the cerebellum of PD patients (Stöger et al., 2017). As this analysis lacks information about where these changes take place in the genome, further experiments will need to shed light on the cause, and examine whether elevated 5hmC levels contribute to PD onset or progression or PD is the reason for the aberrant hydroxymethylation. Approaches that can specifically detect 5hmC have been described, e.g., antibody-based techniques or oxidative bisulfite sequencing (Booth et al., 2012; Skvortsova et al., 2017). These efforts make it likely that methods that discriminate between 5mC and 5hmC will be on hand in the future.

For now, more epigenetic studies are required, particularly ones conducted in different populations, to expand the currently available database of DNA methylation in PD-associated genes. Importantly, a more accurate consensus needs to be reached on the benefit of peripheral blood samples versus brain samples. For the DNA methylation status of a specific gene promoter, such as *SNCA* or other PD-associated genes to be authenticated as a reliable biomarker of PD status, a significant number of studies reporting consistent results will be needed. In this context, careful analysis of Levodopa treatment effects on *SNCA* DNA methylation offers the prospect that in the not-so-distant future a reliable DNA methylation biomarker in PD with high sensitivity and specificity will be available (Schmitt et al., 2015). With the growing interest in research on the interdependency between nutrition and epigenetics, in the future we will get a better understanding of what effects nutritional factors have on DNA methylation and what their involvement is in diseases like PD.

Epigenetic modifications may prove to be the missing link between environmental risk factors and the development of PD. Epigenetic variances between individuals could help us to explain the striking clinical differences observed in the age of onset and progression of sporadic PD, and may open the way for rational therapeutic intervention targeting DNA methylation modifications associated with this disorder.

## Author Contributions

EM-M, KM, AS-C, JS-P, PV-C, and OA-C wrote the manuscript.

## Conflict of Interest Statement

The authors declare that the research was conducted in the absence of any commercial or financial relationships that could be construed as a potential conflict of interest.
